# Supplementary material for: HPV Vaccination in the U.S. Midwest: Barriers and Facilitators of Initiation and Completion in Adolescents and Young Adults
Source: Vaccines (Basel). 2025 Nov 20;13(11):1175. doi: 10.3390/vaccines13111175 (PMC12656891; doi:10.3390/vaccines13111175)
Supplement: Supplementary file 1 [file vaccines-13-01175-s001.zip › vaccines-3949126-supplementary.pdf]

Table S1. Multinomial logistic regression model for predicting HPV vaccination uptake among teenagers and young adults in the Midwest region of the US, among those with unknown vaccination status.

Predictor	*Category*	*Odds Ratio Estimates*	*95% Wald Confidence Limits*	
Have you ever heard about cervical, anal, penile, vaginal, vulva, or oropharynx cancer?	Yes, vs No	0.58	0.40	0.83
Have you ever heard about the HPV vaccine?	Yes, vs No	1.34	0.97	1.87
I am concerned about vaccine side effects	Disagree vs Neutral	0.96	0.63	1.44
I am concerned about vaccine side effects	Agree vs Neutral	0.99	0.69	1.43
Vaccine is one way that I can ensure good health	Disagree vs Neutral	1.10	0.68	1.77
Vaccine is one way that I can ensure good health	Agree vs Neutral	1.60	1.07	2.37
My doctor/health care provider recommended me to receive HPV vaccine	Disagree vs Neutral	0.60	0.39	0.91
My doctor/health care provider recommended me to receive HPV vaccine	Agree vs Neutral	0.50	0.31	0.80
My family member recommends/ supports me to receive HPV vaccine	Disagree vs Neutral	0.45	0.30	0.70
My family member recommends/ supports me to receive HPV vaccine	Agree vs Neutral	0.94	0.58	1.51
I believe that HPV vaccine is safe	Disagree vs Neutral	0.76	0.44	1.34
I believe that HPV vaccine is safe	Agree vs Neutral	1.05	0.67	1.64
I believe that HPV vaccine is effective	Disagree vs Neutral	0.91	0.52	1.61
I believe that HPV vaccine is effective	Agree vs Neutral	1.37	0.88	2.14
My health insurance does not cover the HPV vaccine	Disagree vs Neutral	0.81	0.55	1.21
My health insurance does not cover the HPV vaccine	Agree vs Neutral	0.65	0.41	1.05
I am too young for getting vaccination	Disagree vs Neutral	0.67	0.45	0.99
I am too young for getting vaccination	Agree vs Neutral	0.66	0.43	1.03
I believe that cervical, oropharyngeal, vaginal, vulvar, penile, anal, and rectal cancers are serious	Disagree vs Neutral	1.54	0.88	2.69
I believe that cervical, oropharyngeal, vaginal, vulvar, penile, anal, and rectal cancers are serious	Agree vs Neutral	1.61	1.08	2.41

If I knew a woman with cervical cancer, I would be motivated to get the HPV vaccine	Yes, vs No	0.80	0.57	1.12

Table S2. Multinomial logistic regression model for predicting HPV vaccination uptake among teenagers and young adults in the Midwest region of the US by racial groups.

Predictor	Category	Vaccination status vs Unvaccinated	NH White n=682			NH Black/AA n=272			Hispanic or Latino n= 223			Other* n=129		
			OR	95% CI		OR	95% CI		OR	95% CI		OR	95% CI	
Have you ever heard about cervical, anal, penile, vaginal, vulva, or oropharynx cancer?	Yes vs No	Initiated vaccination	0.72	0.30	1.72	0.29	0.09	0.93	0.81	0.09	7.39	7.52	1.20	47.18
		Fully vaccinated	0.93	0.45	1.93	0.64	0.25	1.59	0.72	0.24	2.17	0.08	0.01	0.48
		Unknown	0.57	0.32	1.02	0.32	0.15	0.70	0.53	0.22	1.30	0.19	0.04	0.97
Have you ever heard about the HPV vaccine?	Yes vs No	Initiated vaccination	1.71	0.79	3.69	3.42	1.06	11.01	4.04	0.46	35.82	1.24	0.92	1.67
		Fully vaccinated	3.02	1.60	5.71	1.98	0.84	4.67	1.94	0.71	5.32	1.08	0.87	1.35
		Unknown	1.36	0.83	2.24	1.79	0.86	3.73	0.54	0.23	1.25	2.22	0.27	18.38
I am concerned about vaccine side effects	Disagree vs Neutral	Initiated vaccination	0.83	0.36	1.92	1.53	0.39	6.04	0.57	0.08	4.34	1.59	0.34	7.37
		Fully vaccinated	1.13	0.59	2.15	0.67	0.22	1.99	1.06	0.35	3.22	1.71	0.49	5.93
		Unknown	0.93	0.51	1.71	1.01	0.38	2.67	0.44	0.15	1.25	1.29	0.33	5.10
	Agree vs Neutral	Initiated vaccination	0.59	0.26	1.35	0.54	0.14	2.07	1.71	0.26	11.18	0.88	0.27	2.81
		Fully vaccinated	0.45	0.23	0.87	0.37	0.14	0.99	0.93	0.32	2.73	1.07	0.13	8.66
		Unknown	1.02	0.59	1.77	0.92	0.40	2.14	1.22	0.48	3.09	2.18	0.41	11.62
Vaccine is one way that I can ensure good health	Disagree vs Neutral	Initiated vaccination	0.87	0.33	2.29	0.06	0.01	0.42	12.13	0.40	366.78	2.23	0.52	9.70
		Fully vaccinated	0.81	0.34	1.93	0.29	0.09	0.99	1.59	0.37	6.71	1.10	0.14	8.84

		Unknown	0.90	0.43	1.86	0.81	0.31	2.14	1.60	0.43	5.98	0.96	0.17	5.42
	Agree vs Neutral	Initiated vaccination	0.44	0.19	1.03	0.40	0.12	1.34	7.95	0.56	113.59	1.33	0.33	5.34
		Fully vaccinated	0.68	0.34	1.35	0.43	0.16	1.16	0.87	0.28	2.69	0.83	0.03	22.36
		Unknown	1.56	0.86	2.84	1.44	0.60	3.42	1.70	0.63	4.59	1.94	0.20	18.39
My doctor/health care provider recommended me to receive HPV vaccine	Disagree vs Neutral	Initiated vaccination	0.42	0.14	1.25	0.76	0.19	3.02	1.34	0.16	11.57	1.50	0.22	10.42
		Fully vaccinated	0.59	0.25	1.42	0.45	0.13	1.59	0.33	0.10	1.13	2.43	0.53	11.11
		Unknown	0.69	0.36	1.33	1.21	0.46	3.18	0.31	0.11	0.86	0.65	0.04	11.10
	Agree vs Neutral	Initiated vaccination	1.97	0.74	5.23	1.10	0.23	5.16	0.22	0.02	1.90	0.18	0.02	1.39
		Fully vaccinated	1.54	0.73	3.27	2.29	0.76	6.95	1.05	0.28	3.89	0.10	0.02	0.53
		Unknown	0.58	0.29	1.18	0.70	0.24	2.00	0.29	0.08	1.05	0.52	0.05	5.15
My family member recommends/ supports me to receive HPV vaccine	Disagree vs Neutral	Initiated vaccination	0.70	0.25	1.99	0.93	0.22	3.86	0.13	0.01	1.50	0.97	0.15	6.18
		Fully vaccinated	0.31	0.13	0.73	0.41	0.12	1.40	0.23	0.06	0.85	0.93	0.08	11.42
		Unknown	0.37	0.19	0.73	0.28	0.11	0.73	0.39	0.13	1.13	1.66	0.22	12.59
	Agree vs Neutral	Initiated vaccination	2.04	0.76	5.50	1.05	0.23	4.89	1.30	0.16	10.29	1.16	0.22	6.17
		Fully vaccinated	2.43	1.14	5.18	1.68	0.59	4.82	0.89	0.25	3.23	2.34	0.26	21.17
		Unknown	0.98	0.47	2.04	0.64	0.23	1.77	0.76	0.23	2.57	12.16	1.97	75.08
I believe that HPV vaccine is safe	Disagree vs Neutral	Initiated vaccination	3.36	1.07	10.57	6.37	1.26	32.31	0.37	0.01	22.46	3.33	0.59	18.92
		Fully vaccinated	1.42	0.49	4.17	6.36	1.58	25.64	0.25	0.04	1.74	2.10	0.14	32.58
		Unknown	0.88	0.35	2.24	0.88	0.29	2.68	0.72	0.14	3.62	5.96	0.66	53.89

	Agree vs Neutral	Initiated vaccination	1.10	0.41	3.00	8.96	1.73	46.45	1.83	0.23	14.45	0.69	0.10	4.88
		Fully vaccinated	0.81	0.37	1.76	8.58	2.43	30.32	0.62	0.18	2.10	7.37	0.70	77.78
		Unknown	0.82	0.41	1.64	1.74	0.61	4.98	0.75	0.24	2.37	1.58	0.22	11.66
I believe that HPV vaccine is effective	Disagree vs Neutral	Initiated vaccination	0.43	0.12	1.59	0.15	0.03	0.86	12.81	0.47	348.75	0.60	0.11	3.20
		Fully vaccinated	0.61	0.17	2.23	0.68	0.17	2.76	1.85	0.31	11.23	0.12	0.00	3.96
		Unknown	0.71	0.26	1.94	0.89	0.28	2.82	0.80	0.20	3.27	0.42	0.04	4.68
	Agree vs Neutral	Initiated vaccination	0.86	0.35	2.13	0.13	0.03	0.65	3.60	0.28	46.78	1.53	0.22	10.50
		Fully vaccinated	2.94	1.40	6.17	0.82	0.27	2.51	2.52	0.66	9.58	0.15	0.01	2.03
		Unknown	1.37	0.71	2.65	0.97	0.36	2.58	2.86	0.86	9.56	0.76	0.10	6.03
My health insurance does not cover the HPV vaccine	Disagree vs Neutral	Initiated vaccination	0.98	0.43	2.25	0.60	0.15	2.36	6.05	0.71	51.79	3.12	0.51	19.12
		Fully vaccinated	1.66	0.88	3.16	0.49	0.18	1.37	1.59	0.52	4.89	8.46	0.92	77.96
		Unknown	0.71	0.39	1.29	0.73	0.30	1.74	1.82	0.64	5.19	0.85	0.17	4.21
	Agree vs Neutral	Initiated vaccination	1.12	0.43	2.91	4.31	1.02	18.23	8.47	0.69	103.84	1.21	0.28	5.21
		Fully vaccinated	1.19	0.53	2.71	1.87	0.57	6.07	0.39	0.11	1.41	13.98	0.86	227.84
		Unknown	0.52	0.25	1.08	0.95	0.31	2.94	0.51	0.16	1.62	2.56	0.27	23.97
I am too young for getting vaccination	Disagree vs Neutral	Initiated vaccination	1.19	0.50	2.85	0.89	0.22	3.65	3.61	0.55	23.83	1.45	0.18	11.67
		Fully vaccinated	1.22	0.63	2.38	0.64	0.23	1.79	1.40	0.48	4.05	1.64	0.17	15.43
		Unknown	0.59	0.33	1.07	0.57	0.23	1.38	1.24	0.45	3.40	1.14	0.22	6.00
	Agree vs Neutral	Initiated vaccination	1.91	0.69	5.26	0.76	0.16	3.69	<0.001	<0.001	>999.99	0.52	0.12	2.22

		Fully vaccinated	1.07	0.44	2.61	0.23	0.06	0.86	0.93	0.28	3.18	1.21	0.11	13.27
		Unknown	0.95	0.48	1.90	0.30	0.10	0.87	1.68	0.57	4.99	0.19	0.03	1.41
I believe that cervical, oropharyngeal, vaginal, vulvar, penile, anal, and rectal cancers are serious	Disagree vs Neutral	Initiated vaccination	6.81	2.02	23.00	17.07	2.33	124.99	2.77	0.10	74.75	0.32	0.01	10.10
		Fully vaccinated	1.54	0.55	4.35	1.58	0.38	6.56	0.93	0.19	4.47	1.52	0.14	16.76
		Unknown	1.24	0.50	3.07	2.03	0.61	6.83	1.01	0.26	3.96	3.25	0.36	29.33
	Agree vs Neutral	Initiated vaccination	3.29	1.17	9.30	2.61	0.56	12.22	1.82	0.09	35.43	0.37	0.03	4.21
		Fully vaccinated	1.50	0.72	3.14	0.53	0.19	1.48	0.88	0.28	2.80	2.09	0.34	13.05
		Unknown	2.54	1.40	4.63	1.52	0.61	3.79	0.62	0.24	1.65	2.66	0.51	13.78
If I knew a woman with cervical cancer, I would be motivated to get the HPV vaccine	Yes vs No	Initiated vaccination	1.90	0.90	4.02	6.48	1.99	21.07	62.38	2.89	>999.99	0.20	0.03	1.58
		Fully vaccinated	1.71	0.96	3.04	2.21	0.95	5.17	3.15	1.20	8.29	0.62	0.13	2.92
		Unknown	0.74	0.45	1.22	0.59	0.27	1.27	1.44	0.61	3.38	0.65	0.17	2.48

*American Indian or Alaska native, Asian, Native Hawaiian or Pacific Islander and Other

Figure S1. Adjusted Odds Ratios by HPV vaccination uptake among teenagers and young adults in the Midwest region of the US by racial groups.

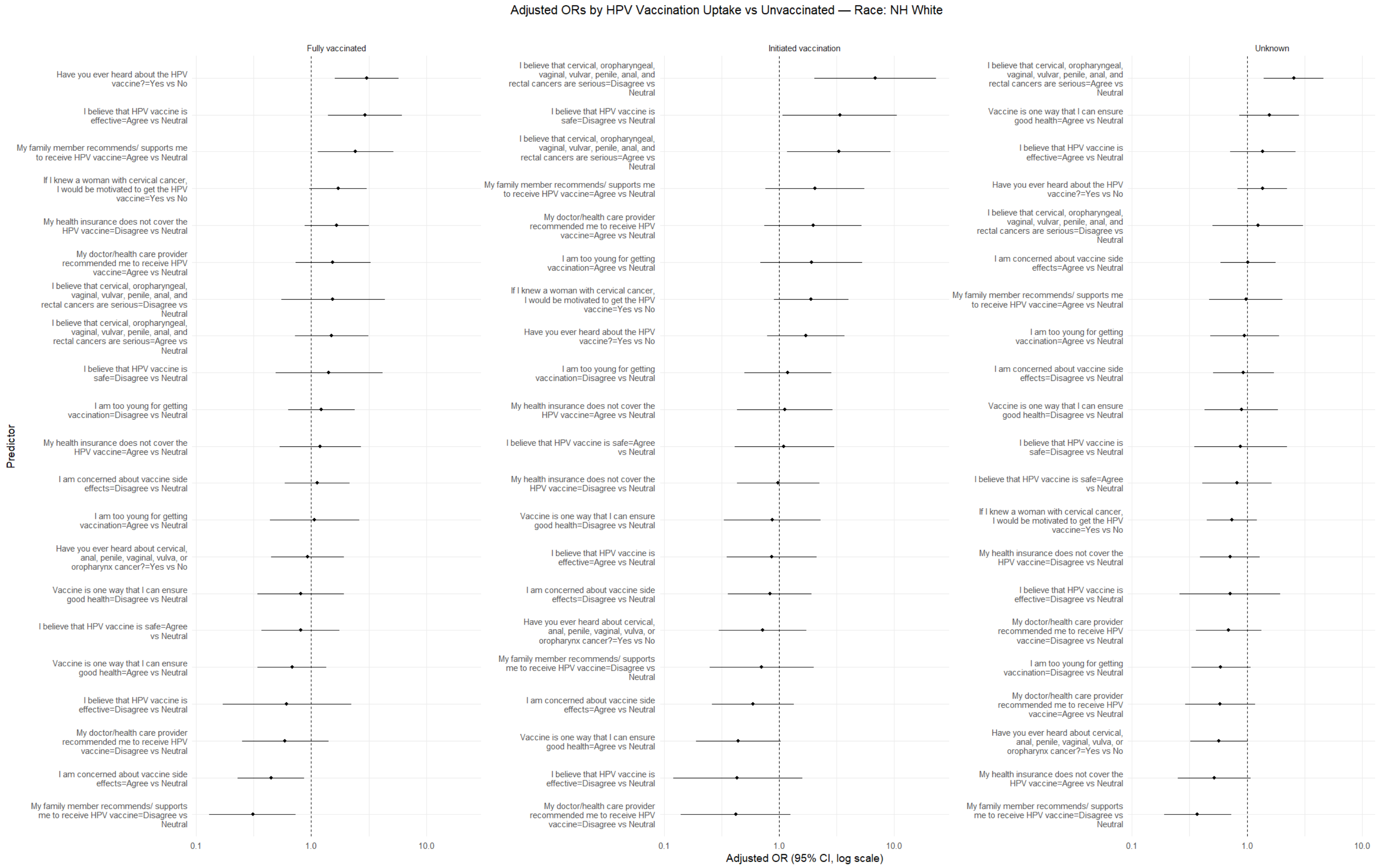

Values to the right of 1 on the x-axis represent increased odds of vaccination. Values to the left of 1 represent decreased odds of vaccination.

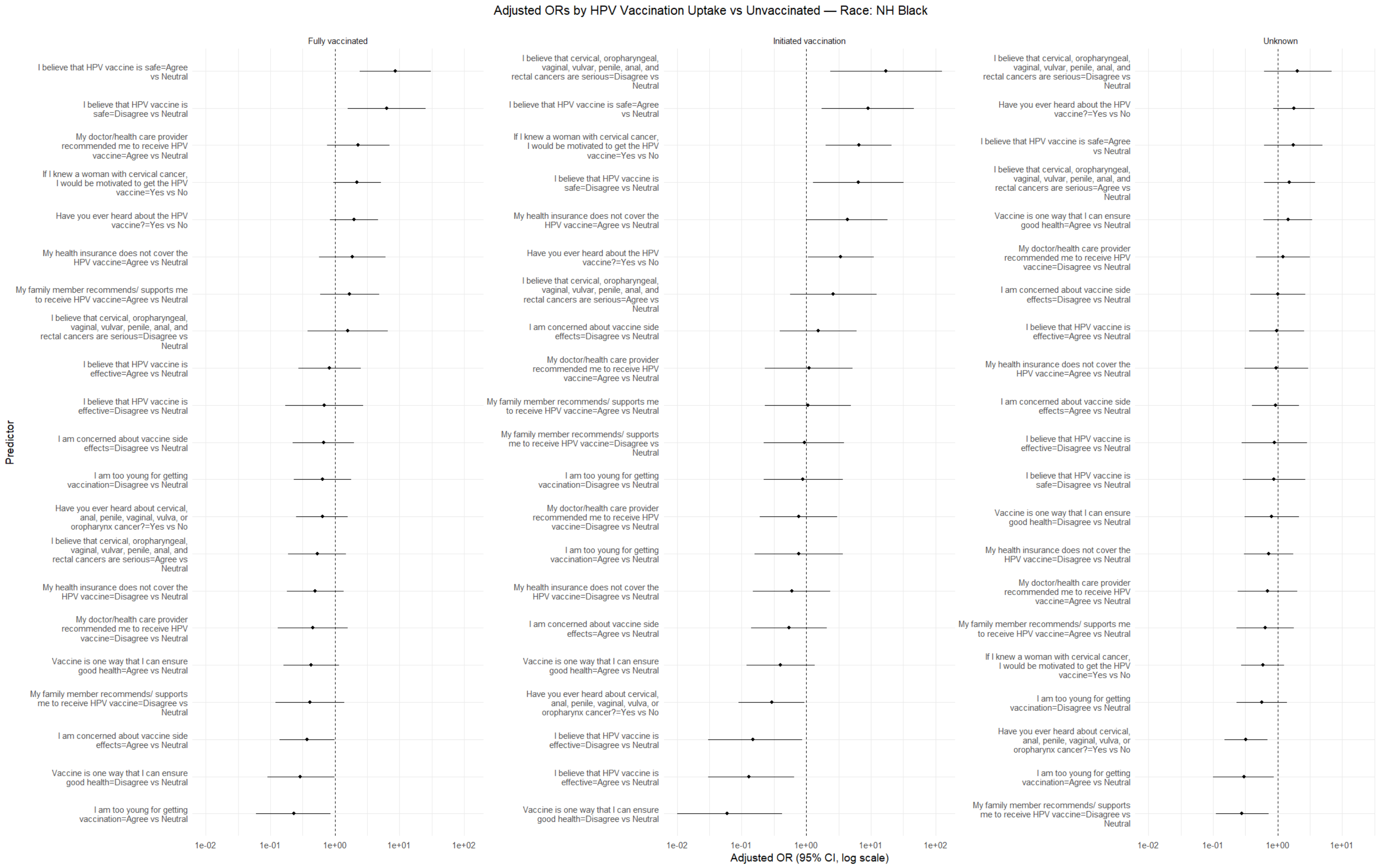

Values to the right of 1 on the x-axis represent increased odds of vaccination. Values to the left of 1 represent decreased odds of vaccination.

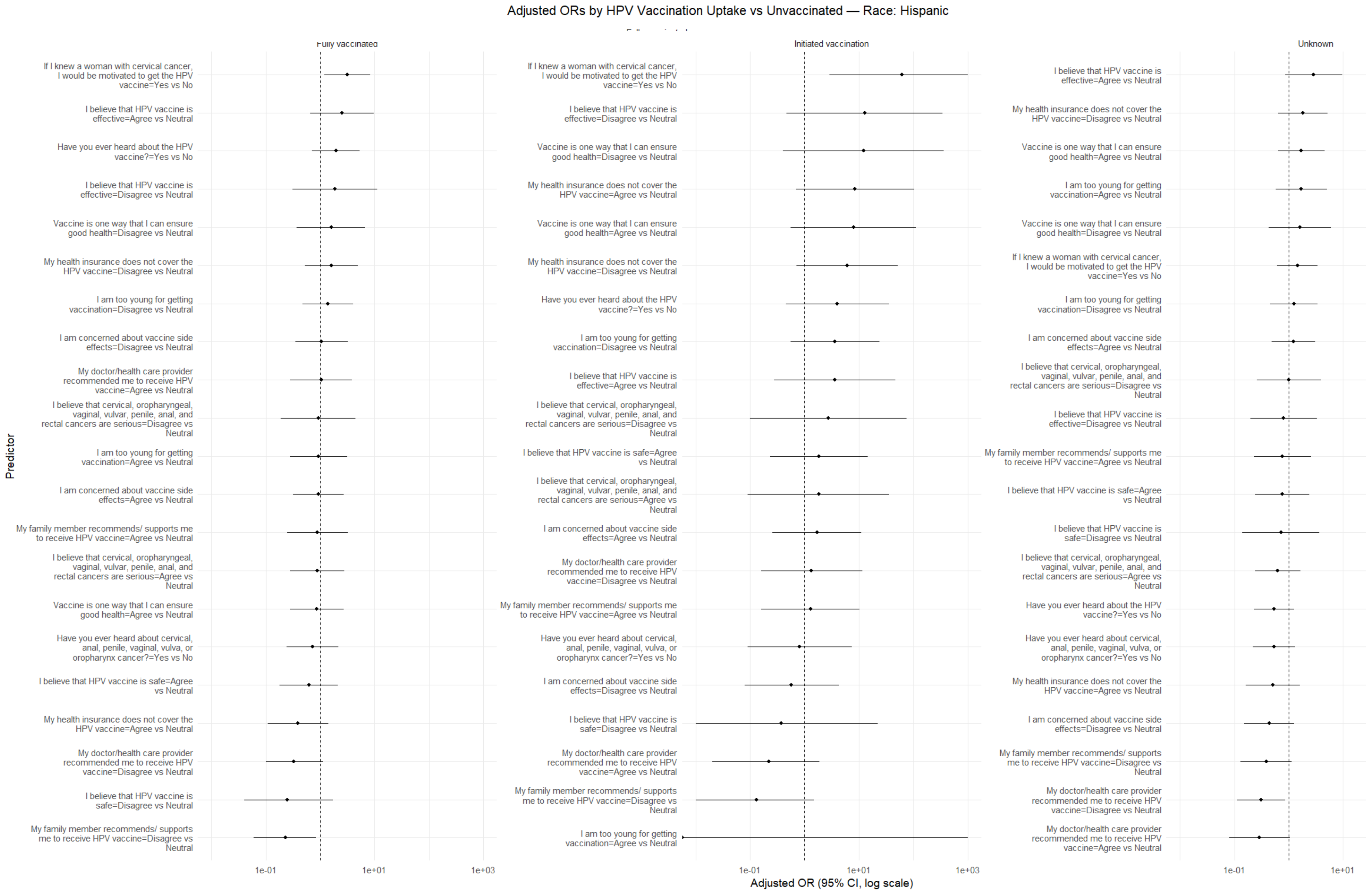

Values to the right of 1 on the x-axis represent increased odds of vaccination. Values to the left of 1 represent decreased odds of vaccination.

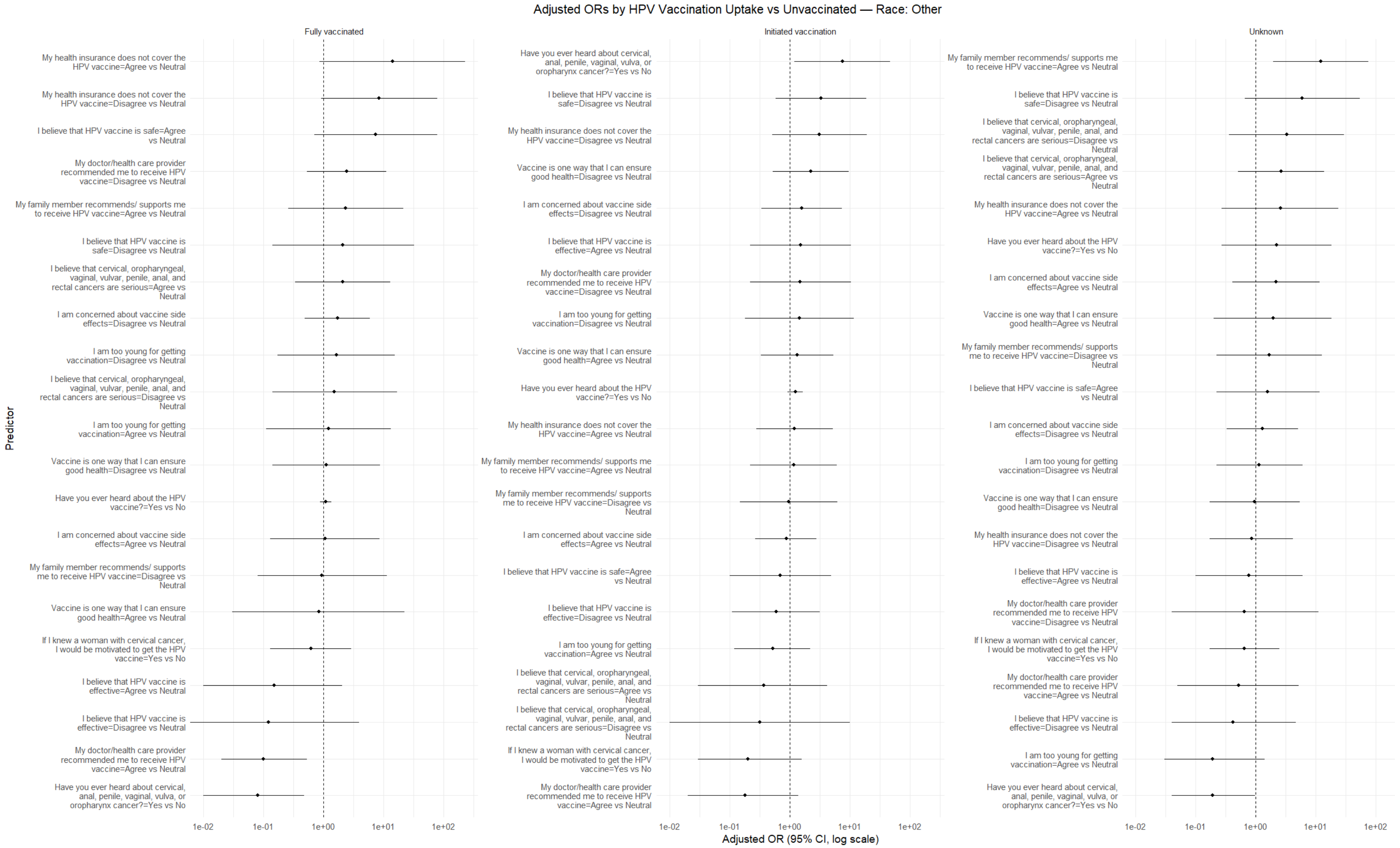

Values to the right of 1 on the x-axis represent increased odds of vaccination. Values to the left of 1 represent decreased odds of vaccination.
